# Cardiovascular risk in young healthcare workers: results from a cross-sectional study

**DOI:** 10.1093/eurpub/ckac130.131

**Published:** 2022-10-25

**Authors:** V Imeshtari, A Imeshtari, G La Torre

**Affiliations:** Department of Public Health and Infectious Diseases, Sapienza University of Rome, Rome, Italy

## Abstract

**Background:**

Cardiovascular diseases represent the main cause of mortality worldwide. While cardiovascular risk (CVR) has decreased in grown adults and elderly in the last years due to innovative therapies and prevention, it seems to be rising among young adults. The aim of our study was to map the 10-year CVR in healthcare workers (HCWs) at the teaching hospital Policlinico Umberto I of Rome and identify possible determinants in order to design and implement preventive strategies.

**Methods:**

A cross-sectional study was carried out between January 2019 and July 2020. 525 HCWs aged 20-40 years were recruited. All participants underwent, after informed consent, medical history collection, physical examination and blood tests. CVR was measured using Framingham Risk Score (FRS) and CUORE score (CVR score developed by the Italian National Institute of Health). Univariate, bivariate and multivariate analysis were performed.

**Results:**

CVR evaluated with FRS correlated positively with age (β = 0.104, p < 0.001), being a shift worker (β = 0.06, p = 0.037), and negatively with female gender (β= -0.757, p < 0.001). No differences were found between being a doctor or a nurse. CVR evaluated with CUORE score correlated positively with age (β = 0.698, p < 0.001), and negatively with female gender (β= -0.332, p < 0.001) and being a doctor (β= -0.220, p < 0.001). Inferential analysis showed low correlation between FRS and CUORE Score (R2=0.340).

**Conclusions:**

Our study demonstrates that females have a lower CVR among HCWs. On the contrary, 15% of male HCWs show a CVR above the average. FRS and CUORE score indicate that advancing age determines an increase in CVR. There is a low correlation between the scores used, in fact CUORE score underestimates the CVR of shift workers while it is known that this particular category is at higher risk.

**Key messages:**

- Some of the HCWs have higher risk of cardiovascular events despite their young age. Further research is needed in order to design appropriate prevention strategies.
- FRS is more accurate in the prediction of CVR among HCWs compared to CUORE score.

